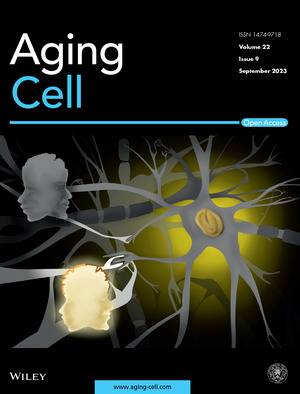# Featured Cover

**DOI:** 10.1111/acel.13992

**Published:** 2023-09-12

**Authors:** Tanita Frey, Tomonari Murakami, Koichiro Maki, Takumi Kawaue, Naoki Tani, Ayaka Sugai, Naotaka Nakazawa, Kei‐ichiro Ishiguro, Taiji Adachi, Mineko Kengaku, Kenichi Ohki, Yukiko Gotoh, Yusuke Kishi

## Abstract

Cover legend: The cover image is based on the Research Article *Age‐associated reduction of nuclear shape dynamics in excitatory neurons of the visual cortex* by Tanita Frey et al., https://doi.org/10.1111/acel.13925